# Comparative Evaluation of Conventional and Molecular Methods for the Diagnosis of Onychomycosis

**DOI:** 10.7759/cureus.112728

**Published:** 2026-07-15

**Authors:** Aditi Dey, Shikha Paul, Sadia Afroz, Najmun Nahar Dina, Shuvro Roy, Shatabdi Mallick, Moshabha Akter Anika, Samah Binte Latif, Raisa Enayet Badhan

**Affiliations:** 1 Microbiology, Community Based Medical College Bangladesh, Dhaka, BGD; 2 Microbiology, Sir Salimullah Medical College, Dhaka, BGD; 3 Laboratory Medicine, National Institute of Traumatology and Orthopaedic Rehabilitation, Dhaka, BGD; 4 Paediatrics, 250 Bed General Hospital, Brahmanbaria, Brahmanbaria, BGD; 5 Microbiology, Khulna Medical College, Khulna, BGD; 6 Microbiology, Directorate General of Health Services, Dhaka, BGD; 7 Microbiology, Dhaka Community Medical College, Dhaka, BGD; 8 Microbiology, National Institute of Burn and Plastic Surgery, Dhaka, BGD

**Keywords:** acridine orange fluorescence microscopy, fungal culture, koh microscopy, onychomycosis, pas stain, pcr

## Abstract

Background

Onychomycosis is a common fungal infection of the nail that often presents diagnostic challenges. Conventional laboratory methods such as microscopy and culture may show limited sensitivity, leading to delayed diagnosis and treatment. Molecular methods such as polymerase chain reaction (PCR) may improve diagnostic accuracy.

Objectives

To compare different diagnostic methods for onychomycosis by evaluating their sensitivity, specificity, positive predictive value (PPV), and negative predictive value (NPV).

Methods

This cross-sectional descriptive study was conducted from July 2023 to June 2024 at Sir Salimullah Medical College (SSMC), Dhaka. A total of 139 nail samples were collected from patients with clinically suspected onychomycosis attending the outpatient department of Dermatology and Venereology. The samples were examined by potassium hydroxide (KOH) mount microscopy, fungal culture, acridine orange stain fluorescence microscopy, periodic acid-Schiff (PAS) stain, and PCR. Laboratory investigations were conducted in the Department of Microbiology at SSMC and at Bangladesh Medical University (BMU). Culture was considered the gold standard, and the diagnostic performances of other methods were evaluated.

Results

Among the 139 samples, KOH mount microscopy detected 65 (46.79%) cases, PAS stain detected 31 (22.30%) cases, acridine orange fluorescence microscopy detected 68 (48.92%) cases, and PCR detected 98 (70.50%) cases. Dermatophytes were isolated in 70 (50.36%) cases and *Candida albicans* in 11 (8%) cases, with *Trichophyton mentagrophytes* being the most common species. Compared with culture, PCR demonstrated the highest sensitivity (97.53%) and NPV (95.12%), while PAS stain showed the highest specificity (96.55%).

Conclusion

PCR demonstrated superior sensitivity compared with conventional diagnostic methods and may facilitate early and accurate diagnosis of onychomycosis when applied directly to clinical samples.

## Introduction

Onychomycosis is a common fungal infection of the nails and represents approximately 50% of all nail diseases worldwide. It affects about 5.5% of the global population and constitutes a significant public health concern [1]. The infection can involve different parts of the nail unit, including the nail bed, nail plate, nail matrix, cuticle, and surrounding nail folds [2].

Clinically, onychomycosis may present with a variety of manifestations such as nail discoloration, thickening of the nail plate, subungual hyperkeratosis, onycholysis, and nail deformity. These changes may significantly affect the nails' cosmetic appearance and impair their normal function [3]. Patients frequently experience discomfort, pain, and difficulty performing routine activities such as walking or wearing shoes. In addition to the physical symptoms, the disease may also cause social embarrassment and psychological distress [4].

The etiological agents responsible for onychomycosis include dermatophytes, yeasts, and non-dermatophyte molds (NDMs). Dermatophytes are the most common pathogens and account for the majority of toenail infections. Among them, *Trichophyton rubrum *and *Trichophyton mentagrophytes* are the predominant species [5]. The distribution of these pathogens varies depending on geographic location, climate, and individual lifestyle factors [6].

Several laboratory methods are available for the diagnosis of onychomycosis. Direct microscopic examination using potassium hydroxide (KOH) preparation is widely used because it is simple, rapid, and inexpensive. However, the sensitivity of KOH microscopy varies widely, ranging from 23% to 77%, and false-negative results may occur in approximately 5% to 15% of cases [7,8]. To improve the sensitivity of microscopic examination, fluorescent stains such as Calcofluor white and acridine orange may be used, although these techniques require a fluorescence microscope [9].

Fungal culture has traditionally been considered the gold standard for diagnosing onychomycosis because it allows identification of the causative organism. However, fungal culture has relatively low sensitivity, ranging from 30% to 57%, and requires a prolonged incubation period before results are available [8].

Histopathological examination using periodic acid-Schiff (PAS) staining of nail biopsy specimens is another useful diagnostic technique. PAS staining can detect fungal elements within the nail plate and has been reported to have a diagnostic sensitivity ranging from 84% to 100%, which is considerably higher than that of direct microscopy or culture [9].

Molecular diagnostic techniques such as polymerase chain reaction (PCR) have recently emerged as promising tools for the detection of fungal pathogens. PCR can amplify small quantities of fungal DNA and target specific genetic sequences such as subtilisin, DNA topoisomerase II, chitin synthase, and internal transcribed spacer (ITS) regions for accurate identification of fungal species. PCR offers several advantages, including rapid detection, high sensitivity, and improved diagnostic accuracy compared with conventional methods [9].

Considering the limitations of conventional diagnostic methods and the increasing availability of molecular techniques, it is important to evaluate the diagnostic performance of different laboratory methods used in the detection of onychomycosis. Therefore, this study was designed to compare conventional and molecular diagnostic methods for the accurate detection of onychomycosis.

## Materials and methods

This cross-sectional study was conducted in the Department of Microbiology, Sir Salimullah Medical College, Dhaka, Bangladesh, over a period of one year from July 2023 to June 2024. The study population comprised clinically diagnosed cases of onychomycosis attending the outpatient departments of Dermatology and Venereology at Sir Salimullah Medical College, Dhaka, and Mugda Medical College, Dhaka. Eligible patients who fulfilled the inclusion criteria were enrolled consecutively during the study period.

Selection criteria

Patients were selected based on predefined inclusion and exclusion criteria. Both male and female patients of any age presenting with clinical features suggestive of onychomycosis were included in the study. All clinical types of onychomycosis were considered, including distal lateral subungual onychomycosis (DLSO), proximal subungual onychomycosis (PSO), white superficial onychomycosis (WSO), and total dystrophic onychomycosis (TDO). Nails showing one or more characteristic features such as onycholysis, subungual hyperkeratosis, nail discoloration, or nail plate thickening were included. Only individuals who provided informed written consent were enrolled in the study.

Patients who had received topical or systemic antifungal treatment for the present nail condition within the last one month were excluded. In addition, patients with other dermatological diseases associated with nail changes, such as psoriasis, lichen planus, eczema, and atopic dermatitis, were excluded. Individuals with permanent or semi-permanent discoloration of the nail plate due to cosmetic or occupational dye exposure were also excluded from the study.

Sample size determination and sampling technique

Both fingernail and toenail specimens were collected from clinically diagnosed cases of onychomycosis using purposive sampling. The required sample size was determined using the standard formula for estimating a single population proportion, $n = \frac{Z^2pq}{d^2}$, where Z = 1.96 at a 95% confidence level, p = 0.10 (estimated prevalence of onychomycosis according to a previous study [10], q = 0.90, and d = 0.05. The calculated sample size was 138.69, which was rounded up to 139.

Accordingly, a total of 139 nail samples were included in the study. Before specimen collection, each participant was interviewed, and relevant demographic and clinical information was recorded using a pre-designed structured data sheet. Following completion of the laboratory investigations, all collected data were compiled, verified, and prepared for analysis.

Ethical consideration

Informed written consent was obtained from each patient. Ethical approval for this study was obtained from the Institutional Ethics Committee of Sir Salimullah Medical College, Dhaka, Bangladesh (Ref. No. 59-14-1100-031-18-001-23-5420). The study was conducted in accordance with the ethical principles of the Declaration of Helsinki. Written informed consent was obtained from all participants before enrollment.

Laboratory procedure

Sample Collection Procedure

The nail area was first cleaned with sterile cotton soaked in 70% ethyl alcohol to eliminate surface contaminants and allowed to air dry before specimen collection [11]. In DLSO, specimens were obtained from the nail bed using a small curette. In WSO, infected nail debris was collected using a No. 15 scalpel blade. In PSO, samples were taken from the proximal infected nail area using a scalpel blade. In TDO, abnormal nail plate or bed material was collected using a nail cutter or nail clipper, including the full length of the onycholytic nail plate where necessary.

Microscopic Examination

**Figure 1:**
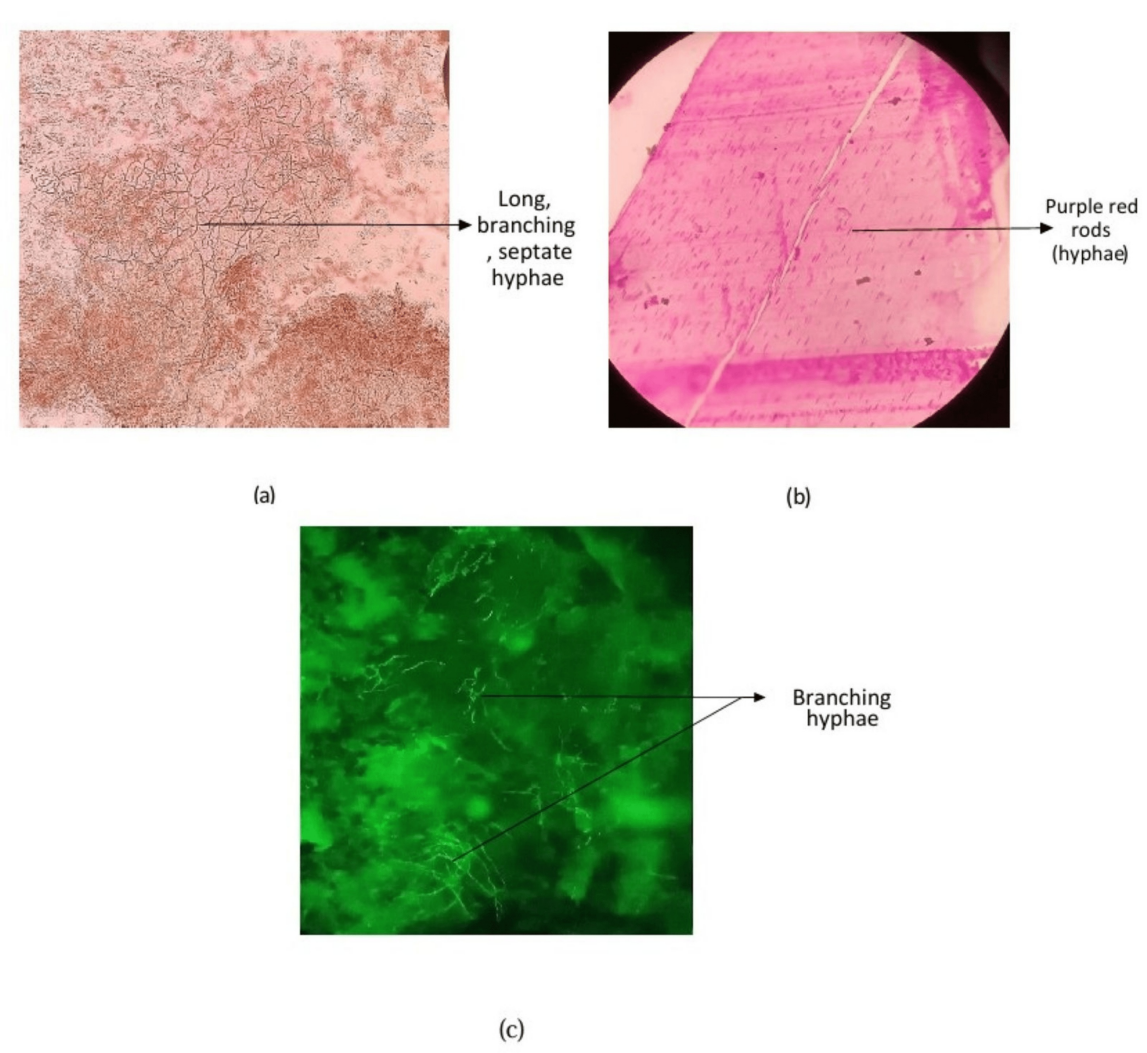
Direct microscopic examination of the nail with KOH mount (a), PAS stain (b), and acridine orange stain fluorescence microscopy (c). Direct microscopic examination of nail specimens using different staining methods. (a) Potassium hydroxide (20% KOH) wet mount showing refractile, branching septate fungal hyphae (black arrows) within the nail keratin, consistent with fungal infection (×100). (b) Periodic acid-Schiff (PAS) stain demonstrating purple-stained fungal hyphae (black arrows) embedded within the nail plate, highlighting fungal cell wall polysaccharides and confirming fungal invasion (×100). (c) Acridine orange fluorescence microscopy showing bright orange fluorescent fungal hyphae (black arrows) against a dark background, facilitating enhanced visualization of fungal elements in the clinical specimen (×100).

Direct microscopic examination was performed using a KOH mount preparation. Dermatophytes were identified under low (10×) and high (40×) power by the presence of long septate, branching hyphae and arthroconidia formed directly from hyphae. Yeasts were identified by the presence of budding yeast cells and pseudohyphae. PAS staining was used as an additional diagnostic method [12]. A positive result was indicated by purple-red dots or thread-like fungal elements present within nail tissue [13]. Acridine orange fluorescent microscopy demonstrated fungal elements as tubular or annular structures with a thin bright green fluorescent rim outlining fungal cell walls (Figure 1) [14].

Culture and Isolation

Culture of dermatophytes was performed using Sabouraud dextrose agar (SDA), the standard medium for fungal isolation. To suppress bacterial contamination, chloramphenicol (0.05 g/L) was incorporated into the medium, while cycloheximide (0.25 g/500 mL) was added to inhibit the growth of rapidly growing NDMs, thereby facilitating the selective isolation of dermatophytes. All clinical specimens were inoculated onto SDA supplemented with chloramphenicol and cycloheximide and incubated for the primary isolation and identification of dermatophytes according to established mycological protocols [15]. Inoculation was performed by placing nail fragments and scrapings directly onto the agar surface using a sterile wire loop after allowing the medium to reach room temperature and dry. Tubes were loosely capped to ensure adequate oxygen availability for fungal growth.

All inoculated media were incubated at 25-28°C and examined daily for up to four weeks. This incubation temperature was selected because dermatophytes exhibit optimal growth and characteristic colony morphology within the range of 25-30°C, facilitating accurate identification. In contrast, incubation at 37°C is not routinely recommended for primary isolation of dermatophytes, as it may suppress and alter colony morphology in some species. Yeast isolates generally produced visible colonies within 24-48 hours at 37°C, whereas dermatophytes required prolonged incubation of up to four weeks before cultures were considered negative.

Microscopic Identification of Cultured Organisms

**Figure 2:**
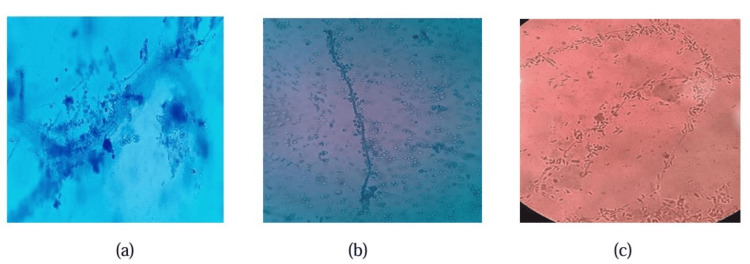
Microscopic view of cluster-shaped Trichophyton mentagrophytes (a), tear-drop-shaped Trichophyton rubrum (b), and ciger-shaped Trichophyton tonsurans (c). Microscopic morphology of dermatophyte isolates following lactophenol cotton blue (LPCB) staining. (a) *T. mentagrophytes* showing characteristic grape-like (cluster-shaped) microconidia arranged along branched hyphae, a typical morphological feature used for species identification (×100). (b) *T. rubrum* demonstrating characteristic tear-drop (pyriform) microconidia borne singly along the sides of septate hyphae (×100). (c) *T. tonsurans* showing characteristic cigar-shaped macroconidia, consistent with the morphological features of the species (×100).

**Figure 3:**
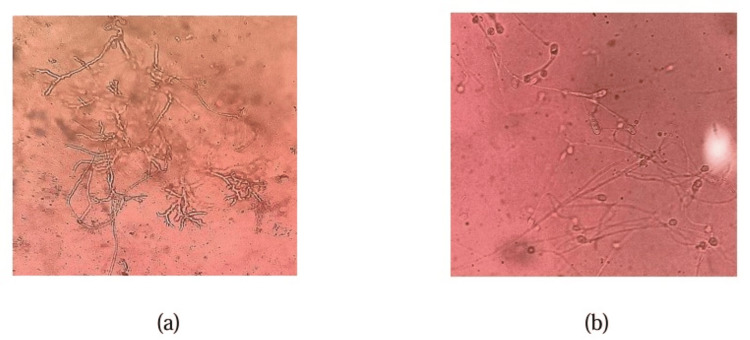
Microscopic view of Trichophyton schoeleinii showing irregular hyphae with lateral and terminal branches (a), and Epidermophyton floccosum showing club-shaped macroconidia (b). Microscopic morphology of dermatophyte isolates following lactophenol cotton blue (LPCB) staining. (a) *T. schoenleinii* showing characteristic irregular, septate hyphae with lateral and terminal branches, a distinctive microscopic feature useful for species identification (×100). (b) *E. floccosum* demonstrating characteristic smooth-walled, club-shaped (clavate) macroconidia, typically multicellular and produced in clusters, consistent with the diagnostic morphology of the species (×100).

Colony morphology and microscopic features were evaluated using lactophenol cotton blue (LPCB) tease mount preparation. A portion of fungal growth was placed on a slide in LPCB stain, teased gently, covered with a coverslip, and examined under low and high power for hyphae, microconidia, macroconidia, and accessory structures. Dermatophytes were further characterized based on species-specific microscopic morphology [16]. Microscopic views of cultured organisms are shown in Figures 2-3.

Yeast Identification

**Figure 4:**
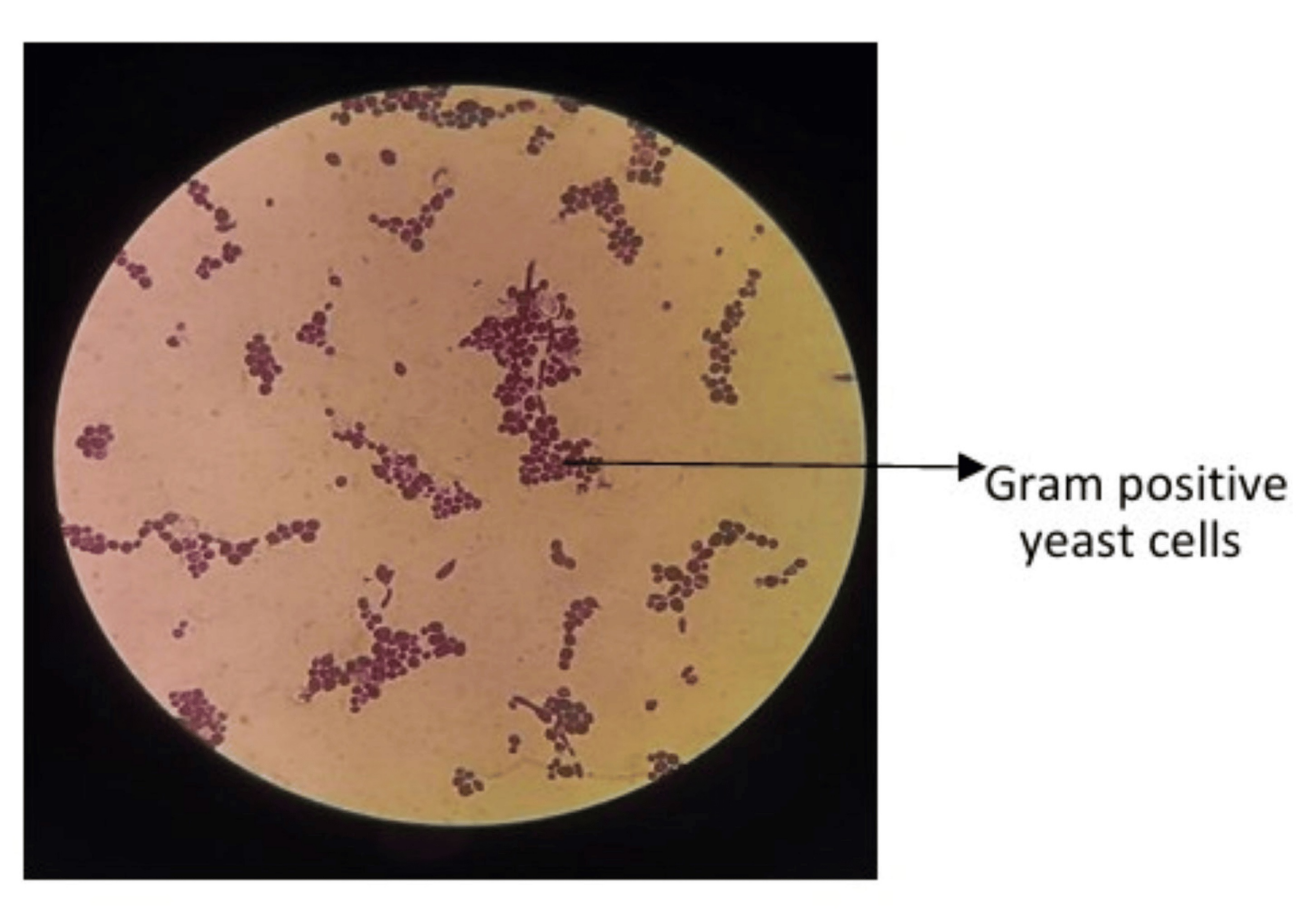
Microscopic view of Candida albicans from culture in Gram stain, 100X. Gram-positive oval budding yeast cells (black arrows), with occasional pseudohyphae, are visible, demonstrating the characteristic microscopic morphology of *C. albicans* (×100, oil immersion). Black arrows indicate the diagnostic budding yeast cells and pseudohyphae.

Wet mount preparation was performed following standard procedures, where *Candida* species appeared as oval to round budding yeast cells, with or without pseudohyphae. Gram staining was also performed, demonstrating Gram-positive budding yeast cells, blastoconidia, and pseudohyphae under microscopy (Figure 4).

Biochemical identification of fungal isolates

Dermatophyte Identification

Biochemical characterization of dermatophyte isolates was performed using urease and hair perforation tests [17]. For the urease test, pure fungal isolates were inoculated onto Christensen’s urea agar (HiMedia Laboratories Pvt. Ltd., Mumbai, India) and incubated at room temperature for 14 days. A change in the medium color from straw to pink was interpreted as a positive result, while the absence of color change was considered negative. *T. mentagrophytes* showed positive urease activity, whereas *T. rubrum*, *Trichophyton schoenleinii*, and *Epidermophyton floccosum* were urease negative.

The hair perforation test was performed using sterilized hair fragments incubated in sterile distilled water supplemented with 10% yeast extract and inoculated with the test fungus [17]. The preparations were incubated at room temperature and examined microscopically after 7, 14, and 28 days using LPCB. Wedge-shaped perforations of the hair shaft were considered positive. *T. mentagrophytes* produced positive hair perforation tests, whereas *T. rubrum* and *E. floccosum* remained negative.

Candida Identification

*Candida* isolates were identified by the germ tube test following the method described by a previous study [18]. Suspected *Candida* colonies were inoculated into 0.5 mL of human serum and incubated at 37°C for two to three hours. A drop of the suspension was examined microscopically for germ tube formation. The presence of a filamentous extension arising from the yeast cell without constriction at its point of origin was considered a true germ tube and indicative of *Candida albicans*, whereas isolates lacking germ tube formation were considered non-albicans *Candida* species.

Polymerase chain reaction (PCR)

PCR amplification was performed using PowerPol 2× PCR Mix with Dye (Cat. No. RK20719; Abclonal Biotechnology Co., Ltd., Wuhan, Hubei, China). Amplification was carried out in a thermal cycler. PCR products were analyzed by agarose gel electrophoresis and visualized using a UV transilluminator.

DNA extraction and amplification

Molecular identification was performed by PCR targeting the ITS region of fungal ribosomal DNA [19]. Nail specimens were cut into small fragments, and genomic DNA was extracted using the QIAamp DNA Mini Kit (QIAGEN GmbH, Hilden, Germany).

Universal fungal primers ITS1 (5′-TCCGTAGGTGAACCTGCGG-3′) and ITS4 (5′-TCCTCCGCTTATTGATATGC-3′) were used for amplification of the ITS region [20].

PCR reactions were prepared in a final volume of 25 µL containing 12.5 µL commercial master mix, 1 µL forward primer, 1 µL reverse primer, 5.5 µL nuclease-free water, and 5 µL DNA template [21].

Amplification was carried out in a thermal cycler with an initial denaturation at 94°C for five minutes, followed by 35 cycles of denaturation at 94°C for one minute, annealing at 56°C for one minute, and extension at 72°C for one minute, with a final extension step at 72°C for seven minutes.

Agarose gel electrophoresis and interpretation

Amplified PCR products were analyzed by electrophoresis on a 1.5% agarose gel prepared in 1× Tris-acetate-ethylenediaminetetraacetic acid (TAE) buffer and stained with ethidium bromide. Electrophoresis was performed at 100 V for 60 minutes alongside a 100 bp DNA ladder. DNA bands were visualized using an INFINITY Vilber Lourmat UV transilluminator (Vilber Lourmat, Marne-la-Vallée, France) and interpreted by comparing their molecular sizes with the marker [22].

**Figure 5:**
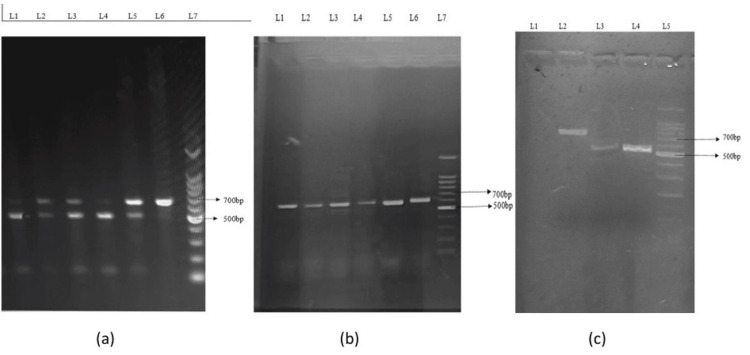
Agarose gel electrophoresis of PCR amplification of the internal transcribed spacer (ITS) gene using universal fungal primers from clinical specimens. (a) Representative agarose gel showing ITS PCR amplification from clinical samples. Lane 7: 1000 bp DNA ladder; lanes 1, 3, and 4: *Trichophyton tonsurans* (550 bp); lane 5: *Trichophyton rubrum* (690 bp); lane 6: *Trichophyton mentagrophytes* (690 bp); lane 2: negative sample with no amplification. (b) Representative agarose gel showing ITS PCR amplification from clinical specimens. Lane 7: 1000 bp DNA ladder; lanes 1, 2, and 3: *T. tonsurans* (550 bp); lanes 4 and 5: *Candida albicans* (595 bp); lane 6: *Trichophyton schoenleinii* (650 bp). (c) Representative agarose gel showing ITS PCR amplification from clinical specimens. Lane 5: 1000 bp DNA ladder; lane 2: *Epidermophyton floccosum* (740 bp); lanes 3 and 4: *C. albicans* (595 bp); lane 1: negative sample with no amplification. The DNA ladder was used as the molecular size marker, and the absence of an amplification band indicates a PCR-negative sample.

Samples showing amplicons within the expected ITS size range (550-740 base pairs (bp)) were considered positive for fungal DNA. Species identification was based on the size of the amplified ITS products: *T. mentagrophytes *and* T. rubrum* (690 bp), *T. tonsurans* (550 bp), *T. schoenleinii *(650 bp), *E. floccosum* (740 bp), and *C. albicans* (595 bp) (Figure 5) [23].

Data analysis

The collected data were analyzed to assess and compare the diagnostic performance of conventional and molecular methods for the diagnosis of onychomycosis. Sensitivity, specificity, positive predictive value (PPV), and negative predictive value (NPV) were calculated for each diagnostic method. Diagnostic accuracy was also determined to evaluate the overall effectiveness of the tests. The results were compared and presented using appropriate descriptive and inferential statistical methods.

## Results

A total of 139 samples of nails were collected from clinically suspected patients of onychomycosis to identify the causative fungal agents of onychomycosis. Different methods were applied to detect fungal agents. The efficacy of applied methods was compared, considering culture as the gold standard.

**Figure 6:**
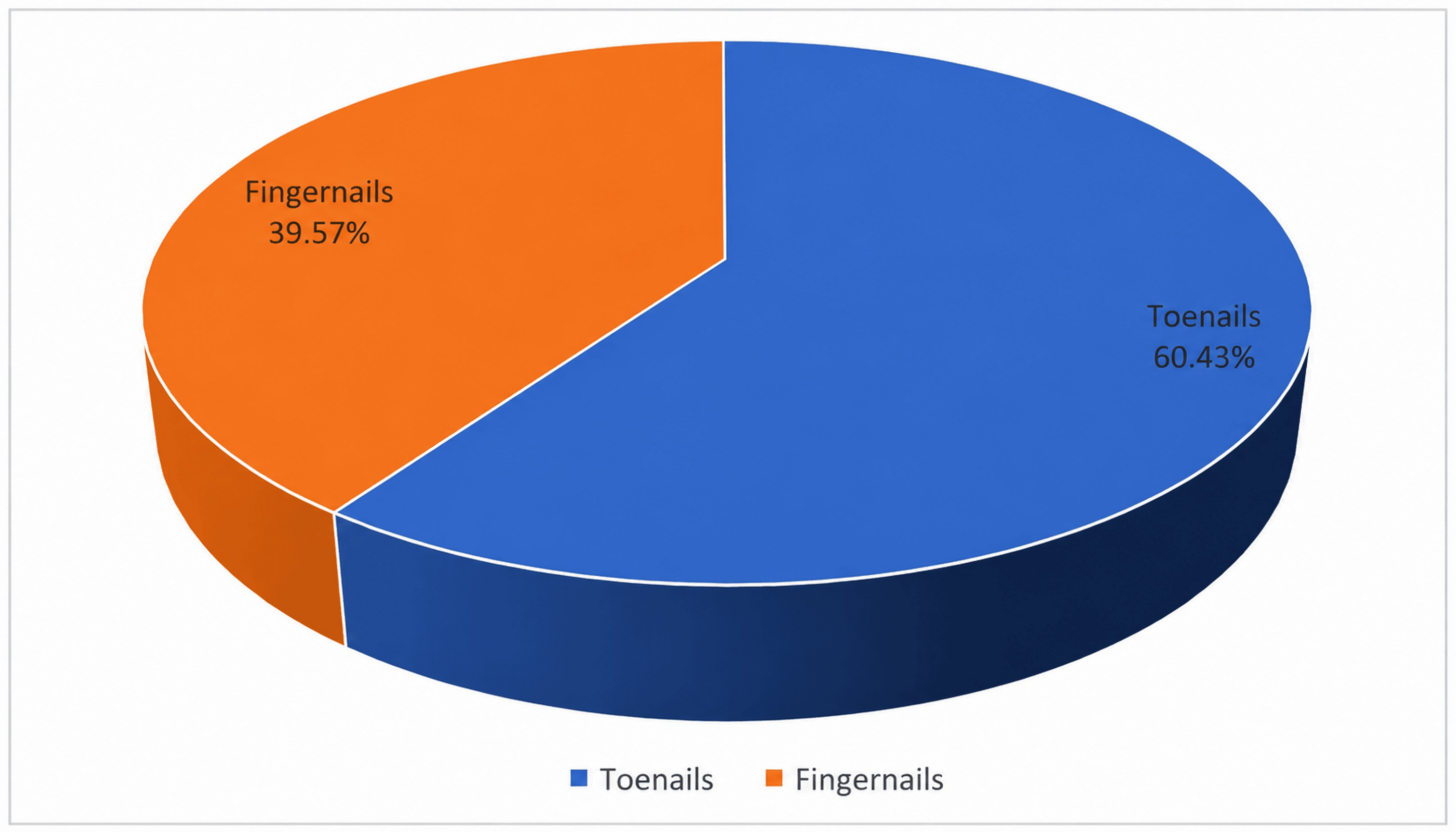
Onychomycosis affecting fingernails and toenails (n = 139).

Out of 139 samples, toenails were the most commonly affected in 60.43% of cases, and fingernails were affected in 39.57% of cases. Toenails were mostly affected with a ratio of 1.53: 1 (toenails: fingernails) (Figure 6).

Table 1 shows the detection of fungal elements in nail specimens by three microscopic diagnostic methods: KOH mount microscopy, PAS stain, and acridine orange fluorescence microscopy. Among the 139 nail samples examined, acridine orange fluorescence microscopy demonstrated the highest detection rate, identifying fungi in 68 (48.92%) specimens, followed closely by KOH mount microscopy, which detected fungi in 65 (46.76%) samples. PAS stain showed the lowest detection rate, identifying fungal elements in 31 (22.30%) specimens. For all three methods, fungal detection was more frequent in toenail specimens than in fingernail specimens. The highest positivity was observed in toenail samples examined by KOH mount microscopy and acridine orange fluorescence microscopy, each detecting fungi in 43 (51.19%) cases.

**Table 1:** Detection of fungi by different microscopic methods according to nail site (n = 139). Values are presented as numbers (percentages). Percentages were calculated based on the total number of specimens from each nail site (toenails, n = 84; fingernails, n = 55). KOH: potassium hydroxide; PAS: periodic acid-Schiff

Nail Sites	KOH Mount Microscopy		PAS Stain		Acridine Orange Fluorescence Microscopy	
	Identified, n (%)	Not Identified, n (%)	Identified, n (%)	Not Identified, n (%)	Identified, n (%)	Not Identified, n (%)
Toenails (n = 84)	43 (51.19)	41 (48.81)	27 (32.14)	57 (67.86)	43 (51.19)	41 (48.81)
Fingernails (n = 55)	22 (40.00)	33 (60.00)	4 (7.27)	51 (92.73)	25 (45.45)	30 (54.55)
Total (n = 139)	65 (46.76)	74 (53.24)	31 (22.30)	108 (77.70)	68 (48.92)	71 (51.08)

Among 139 samples of dermatophytes, NDMs, and *Candida* spp. were isolated in 70 (50.36%), 36 (25.90%), and 11 (8.00%) cases, respectively. Dermatophytes were the most causative fungi isolated by culture. NDM could not be considered a pathogen. So, total fungal isolation was 81 (58.27%).

Table 2 shows the identification of fungal species from cultures by clinical types. Fingernails (55 cases) were affected by *T. mentagrophytes*, *T. rubrum*, and *E. floccosum* in 17 (34%), five (45.45%), and one (25%) case. Toenails (84 cases) were affected by *T. mentagrophytes* in 33 (66%) cases, *T. rubrum* in six (54.55%) cases, and *E. floccosum* in three (75%) cases. *T. tonsurans* and *T. schoeleinii *affected toenails only. *C. albicans *in fingernails, seven (63.64%) cases, and in toenails, four (36.36%) cases.

**Table 2:** Distribution of fungal species isolated by culture among toenail and fingernail specimens (n = 81). Values are expressed as numbers (percentages). Percentages in the toenail and fingernail columns were calculated using the total culture-positive toenail (n = 51) and fingernail (n = 30) isolates, respectively.

Fungal Species	Toenails, n (%) (n = 51)	Fingernails, n (%) (n = 30)	Total, n (%) (n = 81)
Trichophyton mentagrophytes	33 (64.71)	17 (56.67)	50 (61.73)
Trichophyton rubrum	6 (11.76)	5 (16.67)	11 (13.58)
Trichophyton tonsurans	3 (5.88)	0 (0.00)	3 (3.70)
Trichophyton schoenleinii	2 (3.92)	0 (0.00)	2 (2.47)
Epidermophyton floccosum	3 (5.88)	1 (3.33)	4 (4.94)
Candida albicans	4 (7.84)	7 (23.33)	11 (13.58)
Total	51 (100.00)	30 (100.00)	81 (100.00)

Table 3 shows the detection of fungal isolates by PCR. Out of 55 cases of fingernails, 41 (41.84%) fungal isolates, and out of 84 cases of toenails, 57 (58.16%) isolates were identified by PCR. A total of 98 (70.50%) fungal isolates were identified by PCR in both fingernails and toenails.

**Table 3:** Identification of fungal species detected by PCR from clinical types (n= 139). Values are expressed as numbers (percentages). Percentages in the toenail and fingernail columns were calculated within each fungal species. Overall percentages were calculated using the total number of PCR-positive isolates (n = 98).

Fungal Species	Toenails, n (%)	Fingernails, n (%)	Total, n (%)
Trichophyton mentagrophytes	40 (66.67)	20 (33.33)	60 (61.22)
Trichophyton rubrum	6 (54.55)	5 (45.45)	11 (11.22)
Trichophyton tonsurans	3 (75.00)	1 (25.00)	4 (4.08)
Trichophyton schoenleinii	2 (66.67)	1 (33.33)	3 (3.06)
Epidermophyton floccosum	4 (80.00)	1 (20.00)	5 (5.10)
Candida albicans	2 (13.33)	13 (86.67)	15 (15.31)
Total	57 (58.16)	41 (41.84)	98 (100.00)

Table 4 compares the diagnostic performance of KOH mount microscopy, acridine orange fluorescence microscopy, and PAS stain against culture for the diagnosis of onychomycosis. Among the three methods, acridine orange fluorescence microscopy demonstrated the highest sensitivity (74.07%), specificity (86.21%), and NPV (70.42%), indicating superior overall diagnostic performance. PAS stain showed the highest specificity (96.55%) and PPV (93.55%), suggesting excellent ability to confirm fungal infection when positive, although it exhibited the lowest sensitivity (35.80%). KOH mount microscopy demonstrated moderate sensitivity (62.96%) and specificity (75.86%). Overall, acridine orange fluorescence microscopy appeared to be the most balanced diagnostic method when compared with culture.

**Table 4:** Comparison of KOH mount microscopy, acridine orange fluorescence microscopy, and PAS stain with culture in the diagnosis of onychomycosis (n = 139). Culture was considered the reference standard for comparison.

Diagnostic Method	True Positive (TP)	False Positive (FP)	False Negative (FN)	True Negative (TN)	Sensitivity (%)	Specificity (%)	PPV (%)	NPV (%)
Potassium hydroxide (KOH) mount microscopy	51	14	30	44	62.96	75.86	78.46	59.46
Acridine orange fluorescence microscopy	60	8	21	50	74.07	86.21	88.24	70.42
Periodic acid-Schiff (PAS) stain	29	2	52	56	35.80	96.55	93.55	51.85

Table 5 shows a comparison of PCR with culture in the diagnosis of onychomycosis. Out of 98 positive cases by PCR, culture was positive in 79 cases and negative in 19 cases. Two cases were positive by culture but negative by PCR, and 39 cases were negative in both diagnostic tests. The culture was considered the gold standard when PCR was compared to culture sensitivity of PCR was 97.53%, specificity was 67.64%, PPV was 80.61%, and NPV was 95.12%.

**Table 5:** Comparison of PCR with culture in the diagnosis of onychomycosis (N= 139). PPV: positive predictive value; NPV: negative predictive value; PCR: polymerase chain reaction

PCR	Culture Positive	Culture Negative	Total	Sensitivity (%)	Specificity (%)	PPV (%)	NPV (%)
Positive	79	19	98	-	-	-	-
Negative	2	39	41	-	-	-	-
Total/diagnostic accuracy	81	58	139	97.53	67.24	80.61	95.12

Table 6 shows a comparison of the validity test of different methods in the diagnosis of onychomycosis in relation to culture. Culture was taken as the gold standard, and when KOH mount microscopy was compared to culture, the sensitivity of KOH microscopy was 62.96%, and the specificity was 75.86%. When acridine orange stain was compared to culture, the sensitivity of acridine orange stain was 74.07%, and the specificity was 86.21%. When PAS was compared to the culture, the sensitivity of PAS was 35.80%, and the specificity was 96.55%. When PCR was compared to culture, the sensitivity of PCR was 97.53%, and the specificity was 67.24%. PPV of KOH mount, acridine orange stain, PAS, and PCR was found to be 78.46%, 88.24%, 93.55%, and 80.61%, respectively. NPV of KOH mount, acridine orange stain, PAS, and PCR was found to be 59.46%, 70.42%, 51.85%, and 95.12%, respectively. The highest sensitivity was observed in PCR with 97.53%, and the highest specificity was found in PAS with 96.55%.

**Table 6:** Comparison of validity test of different methods in diagnosis of onychomycosis in relation to culture (N = 139). PPV: positive predictive value; NPV: negative predictive value; KOH: potassium hydroxide; PAS: periodic acid-Schiff; PCR: polymerase chain reaction

Diagnostic Test	Sensitivity (%)	Specificity (%)	PPV (%)	NPV (%)
KOH mount microscopy	62.96	75.86	78.46	59.46
Acridine orange fluorescence microscopy	74.07	86.21	88.24	70.42
PAS stain	35.80	96.55	93.55	51.85
PCR	97.53	67.24	80.61	95.12

## Discussion

Fungal nail infection, known as onychomycosis, makes up about half of all nail disorders. This condition can spread and persist, causing both psychological and financial impacts. Therefore, it is essential to have access to reliable laboratory techniques for quickly and accurately identifying the fungi responsible for the infection to administer appropriate treatment and prevention measures. The present study aimed to evaluate and compare the diagnostic performance of different laboratory methods for the diagnosis of onychomycosis.

In this study, onychomycosis more commonly affected toenails in 84 (60.43%) cases than fingernails in 55 (39.57%) cases. This finding was comparable with the studies conducted previously [20,23]. The reason for the higher frequency of fungal infection of toenails was probably open footwear and a lesser concern for the appearance of feet and toenails [24]. DLSO (51.08%) was the most common clinical type in the current study, which was comparable to the findings of previous studies [20,23].

In the present study, 65 out of 139 samples (46.76%) tested positive using KOH mount microscopy. A study showed [23] that 46.08% of cases tested positive using direct microscopy with KOH mount out of 102 samples, which was similar to the findings of the current study. Various studies have reported different detection rates for these methods. Some other studies showed nearly similar findings at 52% and 57.6%, respectively [20,25].

Different studies showed inconsistent results in diagnosing onychomycosis through culture. A study found a higher positivity rate of 70%. Previous studies indicated that fungal culture is more accurate than KOH preparation in identifying the causes of onychomycosis. However, false negatives can occur due to non-viable organisms that do not grow in culture, inaccessibility of the organism in the nail to the culture growth media, previous treatment with antifungal drugs hindering culture growth, inaccuracies in sampling, and the slow growth of dermatophytes in culture media [26]. False positive results in the culture can also occur due to contamination of the culture medium by normal fungal flora of the skin, laboratory contaminants, or saprophytes [25].

In this study, dermatophytes (50.36%) were found to be the most isolated fungus detected by culture in 70 cases out of 139 samples. A similar result was also reported (60%) [23], but the positivity rate was 31.6% in another study [24].

*T. mentagrophytes* (50, 61.73%) was the main fungus responsible for onychomycosis, followed by *T. rubrum* (11, 13.58%) and *C. albicans* (11, 13.58%) in this study. A similar study [27] where they also found *T. mentagrophytes* (59.21%) as the predominant species, followed by *T. rubrum* (40.79%). In another previous study, *T. mentagrophytes* (41.3%) was found main etiological dermatophyte species, followed by *T. rubrum* (17.3%) [28]. The probable cause of this finding may be too much application of the steroid cream on the skin for fungal infection, which also diminishes the immune status of the host and provides a hospitable environment in the skin to survive *T. mentagrophytes* [29]. A study showed *C. albicans* (36.8%) was the most common causative agent of onychomycosis [20].

In this study, NDMs could not be confirmed as pathogens due to the inability to collect repeated samples, and could not be detected in KOH mount microscopy and other tests.

In the current study, out of 139 samples, 31 (22.30%) cases were positive by PAS stain. A previous study showed that out of the 106 samples, PAS stain showed positive results in 30.2% of cases, which was comparable with the current study [13]. The reason for the low detection rate in PAS stain was probably due to the sample being collected from the free edge of the distal nail plate, but the pathogenic fungus was located preferentially in the transition area between the affected and healthy nails.

In the current study, out of 139 samples, 68 (48.92%) cases were positive by acridine orange stain fluorescence microscope. The study conducted previously showed that out of the 204 samples, acridine orange stain fluorescence microscopy showed positive results in 62% of cases, which was comparable with the current study [30].

Molecular biology methods for the identification of fungi directly from specimens are reliable and reproducible compared to other methods [21]. PCR was positive in 98 (70.50%) cases in this study. A study showed that out of 425 samples, 284 (67%) were positive by PCR, with 198 (47%) of the total reported as dermatophyte, 50 (12%) as saprophyte, and 36 (8%) as yeast. This study was comparable to the current study [8].

Considering fungal culture as the cornerstone, this study showed that the most reliable technique for diagnosis of onychomycosis was PCR (97.53%), followed by acridine orange stain fluorescence microscopy (74.07%), and KOH mount microscopy (62.96%). Another study showed that the sensitivity of PCR was 100%, acridine orange stain fluorescence microscopy was 89%, and KOH mount microscopy was 53% [20].

Other previous studies also showed that the sensitivity of PCR, acridine orange stain, and KOH mount was 78%, 97%, and 85%, respectively [30]. In this study, the use of fungal fluorescent staining of nail specimens for the studied patients enhanced the sensitivity of direct examination by 11.11% compared to KOH (62.96%). Also, another study showed that the use of fungal fluorescent staining of nail specimens improved the sensitivity of direct examination by 12% [30].

In this study, the sensitivity of PAS was 35.80%. A study showed that the sensitivity of PAS was 52% [13]. Most of these studies have shown histological examination to be the most sensitive diagnostic test for onychomycosis, although it can take up to one week for preparation and is more expensive than other tests. Furthermore, this method has the drawback of being unable to identify a viable organism [25].

In the present study, the specificity of PCR was 67.24%. A previous study showed the specificity of PCR was 90% [30]. In this study, the specificity of KOH and acridine orange stain was 75.86% and 86.21%, respectively. The study showed that the specificity of KOH and acridine orange stain was 100% and 89%, respectively [21].

Specificity of PAS was 96.55% in this study, and specificity of PAS was 100% in a study [13]. PPV of PCR, KOH mount, acridine orange stain, and PAS was 80.61%, 77.46%, 88.24%, and 93.55%, respectively. A study showed that the PPV of PCR, KOH mount, acridine orange stain, and PAS was 71%, 71%, 78%, and 87%, respectively [20].

NPV of PCR was the highest (95.12%), followed by acridine orange stain (70.42%), KOH mount (59.46%), and PAS stain (51.85%) in the present study. Another study showed that the NPV of PCR, acridine orange stain, KOH mount, and PAS was 100%, 60%, 31%, and 51%, respectively [20].

This study had several limitations. First, it was conducted at a single tertiary care center with a relatively limited sample size, which may restrict the generalizability of the findings to the broader population. Second, the unavailability of reference strains limited species confirmation by comparison, and the absence of terbinafine discs restricted antifungal susceptibility profiling for a commonly used therapeutic agent. Furthermore, PCR-based identification could not differentiate between *T. mentagrophytes* and *T. rubrum*, which represents an important limitation in precise species-level diagnosis. Despite these constraints, the study underscores the value of integrating multiple diagnostic approaches for improved detection of fungal pathogens in nail infections. Future studies incorporating advanced molecular techniques such as PCR-restriction fragment length polymorphism (RFLP) are recommended to achieve more accurate species differentiation and to strengthen the diagnostic framework for onychomycosis.

## Conclusions

In this study, onychomycosis was more frequently detected in toenails than in fingernails. Among the conventional diagnostic methods evaluated, acridine orange fluorescence microscopy demonstrated higher sensitivity (74.07%) and specificity (86.21%) than KOH mount microscopy (62.96% and 75.86%, respectively), while PAS stain showed the highest specificity (96.55%) and PPV (93.55%). PCR exhibited the highest sensitivity (97.53%) and NPV (95.12%) among all methods tested. These findings suggest that PCR is a highly sensitive diagnostic tool for the detection of onychomycosis, whereas PAS stain provides excellent specificity. The combined use of conventional and molecular methods may improve the overall diagnostic accuracy of onychomycosis.
